# The time of your life: mapping the mechanisms behind life tempo judgments

**DOI:** 10.3389/fpsyg.2025.1747171

**Published:** 2026-01-26

**Authors:** Mark J. Landau, Young-Ju Ryu

**Affiliations:** Department of Psychology, University of Kansas, Lawrence, KS, United States

**Keywords:** autobiographical memory, growth, norms, nostalgia, routine, self-determination theory, time perception

## Abstract

Across history and cultures, people have persistently lamented that life seems to move “too fast.” Psychology has approached this impression from multiple angles, yet lacks an integrative account of why autobiographically meaningful periods so often feel fleeting in retrospect. In this review, we examine the psychological mechanisms that shape life-tempo judgments (LTJs)—retrospective assessments of how quickly or slowly personally significant periods are remembered to have passed. LTJs are not literal perceptions of temporal speed nor comparisons to clock time, but flexible judgments made relative to subjective standards and expectations. We synthesize major theoretical accounts of LTJs, ranging from classic “cold” cognitive explanations centered on routine, memory compression, and attention, to “hot” motivational and affective accounts emphasizing personal growth, longing, social performance, and identity-relevant evaluation. Rather than treating these perspectives as competitors, we evaluate their explanatory scope, empirical support, and points of convergence. Recent empirical work reviewed here highlights a consistent pattern: periods remembered as especially meaningful, engaging, or growth-promoting are often the same periods described as having passed most quickly. We suggest that this pattern is unlikely to be explained by any single mechanism, but instead reflects the joint influence of memory structure, motivational comparison standards, affective meaning, and cultural narratives in the construction of lived time. By organizing a fragmented literature on LTJs, this review provides a conceptual scaffold for future theoretical refinement, empirical research, and translational work. Rather than prescribing how to slow life down, we argue that understanding why life feels fast may help clarify what a fast life signifies—and when it reflects loss, fulfillment, or some combination of the two.

## 1 Introduction

Human beings are unusual in their capacity to step back from the here-and-now and form impressions about the passage of their own lives. We track birthdays, mark milestones, narrate chapters, and ask not only *what* happened, but—looking back—*how fast it all seemed to go*. Strikingly, this capacity—often treated as a hallmark of reflective intelligence—appears to be accompanied by a pervasive sense that life is slipping by too quickly.

This complaint dates back at least to the first century BCE, when the poet Virgil observed that “irretrievable time flies,” and was echoed a century later when the Stoic philosopher Seneca wrote *On the Shortness of Life* to console those distressed by life's apparent brevity. Today, the same sentiment surfaces in remarks like “I can't believe I've been working here 10 years,” “One moment our daughter was a baby, and now she's grown,” “Where did November go?” and “My weekend flew by.”

Empirical research confirms that people often recall lived intervals as having passed more quickly than expected. Across cultures, older adults report that their most recent decade passed more quickly than one from their youth (Wittmann and Lehnhoff, 2005; Wittmann and Mella, 2021). People also report the swift passage of shorter intervals, from weeks and months to 4 day holidays (Avni-Babad and Ritov, 2003; Friedman and Janssen, 2010; Wittmann and Lehnhoff, 2005).

We label this phenomenon *life-tempo judgments* (LTJs in what follows)—retrospective assessments of how quickly or slowly an autobiographically meaningful period is remembered to have passed. Such periods can range from relatively brief intervals (e.g., a vacation, a semester) to extended life chapters (e.g., several years or decades). Although this definition encompasses judgments that a period passed quickly or slowly, we concentrate on the experience of “too fast” LTJs.

Despite the familiarity of this experience, psychology lacks a unified account of the mechanisms that shape LTJs. Classic thinkers from Seneca to William James offered penetrating insights, and contemporary theorists have revisited the question (Burdick, 2017; Draaisma, 2004; Wittmann, 2016), yet empirical research remains sparse.

Cognitive and neuroscientific traditions have long researched how people judge the duration and pace of intervals on the scale of milliseconds or seconds (Arstila and Lloyd, 2021). Although it would be convenient to extend these models to explain LTJs, complications arise. Much of this work focuses on *prospective* judgments—cases in which observers know in advance that they will need to estimate the duration of an ongoing interval. Meta-analyses show that prospective and retrospective judgments rely on partly distinct processes (Block and Zakay, 1997; Zakay and Block, 2004), and it is the latter that interests us here. Moreover, relevant studies typically focus on judgments of brief intervals during which participants process stimuli of minimal personal significance (e.g., word lists). Our concern, by contrast, is with judgments of extended, personally meaningful periods. That said, we'll see that some of these models can be fruitfully adapted to the autobiographical domain.

We aim to map explanatory accounts of LTJs, noting where they succeed, where they falter, and what puzzles remain. We group accounts into two broad families. The first family focuses on what social psychologists call “cold” mechanisms—those theorized to operate at a purely cognitive level with minimal inputs from affect or motivation (Kunda, 1999). The second family includes “hot” mechanisms that model affective factors (e.g., valuations of the quality of remembered experience) or motivational factors (e.g., desire to sustain a positively evaluated self-concept).

First, it is important to clarify what LTJs consist of, and how we will refer to them. Although people colloquially express such judgments using speed-metaphorical terms such as *fast*, they are not experiencing temporal velocity in any literal sense. Nor are LTJs made relative to clock time, as people lack direct, veridical access to objectively measured durations (e.g., December has 744 h). Unlike duration judgments studied in psychophysics, LTJs do not presuppose a stable internal metric or a privileged reference interval. Instead, they reflect flexible, meaning-laden comparisons across autobiographical contexts.

More specifically, in forming LTJs, individuals are likely comparing the felt (or recounted or estimated) duration of a target period against a subjective standard for how long that period was expected or desired to feel. These standards are not fixed: depending on context, people may implicitly compare a target period to other life phases, to typical expectations for similar periods, or to idealized or wished-for alternatives. Many of the explanatory accounts reviewed here can be understood as emphasizing different aspects of this comparative process—whether by shaping how richly a period is remembered, altering the standards against which it is evaluated, or both.

Although LTJs involve over- or under-estimation of remembered durations relative to subjective standards, the phenomenological impression that a period passed quickly is commonly described metaphorically in terms of speed, spatial distance (long/short), or music-theoretical terms like our own use of “tempo.” To characterize this historically and culturally widespread phenomenological impression, we use such metaphorical terms interchangeably throughout this manuscript. Crucially, however, the phenomenon under study concerns retrospective, comparative judgments rather than a literal perception of time's rate, speed, or length.^1^ Our aim is to balance conceptual precision with fidelity to both common experience and established usage in the time perception literature.

## 2 Explanatory considerations

Theoretical proposals concerning LTJs are diverse, spanning cognitive, motivational, cultural, and phenomenological traditions. Rather than attempting to adjudicate among these accounts *a priori*, we begin by outlining a small set of explanatory considerations that can help readers compare their respective strengths, limitations, and domains of applicability. These considerations are not intended as necessary conditions for theoretical adequacy, nor as criteria for excluding particular approaches. Instead, they serve as shared lenses for organizing the literature, clarifying what different accounts appear best-suited to explain, and organize directions for future work.

### 2.1 Generativity

We prioritize accounts capable of generating testable hypotheses regarding the situational or dispositional predictors of LTJs. This consideration guided us to selectively sample the literature on individual differences in LTJs. Relevant studies link faster LTJs to individuals with children (Wittmann and Mella, 2021), those who score high in future orientation (Zimbardo and Boyd, 1999) or present-hedonistic tendencies (Jokic et al., 2018), and those who score low in impulsivity (Wittmann et al., 2015) or boredom proneness (Danckert and Allman, 2005), among other individual differences. We feature this work when it sheds light on underlying mechanisms.

### 2.2 Cross-scale applicability

As noted earlier, empirical and anecdotal evidence suggest that people routinely judge both short life periods (e.g., last week) and long life periods (e.g., last decade) as having passed quickly. In light of this evidence, we tentatively present cross-scale applicability as a marker of explanatory strength. For the sake of scientific parsimony, we might prefer accounts with the breadth to explain LTJs across short and long temporal scales.

We acknowledge that this consideration is not uncontroversial. Within cognitive research, scholars have long debated whether judgments of very short and short intervals (e.g., milliseconds vs. hours) are governed by the same or different mechanisms (Block and Zakay, 1997; Hicks and Miller, 1976; Matthews and Meck, 2016; Ornstein, 1969). Further, in other scientific domains, distinct explanatory frameworks operate at different temporal or spatial scales.^2^ Consequently, privileging accounts that span autobiographical scales can prematurely foreclose the possibility that qualitatively distinct mechanisms govern LTJs at different scales. Thus, we do not imply that cross-scale applicability is a necessary condition for explanatory adequacy.

Our motivation for emphasizing this consideration is not a commitment to unification, but a response to a related theoretical gap: there is currently no well-developed framework for identifying where psychologically meaningful boundaries between temporal scales should be drawn. Intuitively, judgments of one day vs. four decades may differ in kind, but it is far less clear how—or whether—such distinctions apply between intermediate intervals (e.g., weeks vs. months; 1 year vs. 5 years). To our knowledge, neither empirical evidence nor formal theory has yet articulated principled criteria for carving autobiographical time into distinct scales with different governing mechanisms.

Accordingly, we present cross-scale applicability not as an evaluative standard, but as a heuristic preference—one that reflects current theoretical uncertainty rather than a settled conclusion about how autobiographical time perception should be modeled.

### 2.3 Historical generalizability

Complaints that life seems to slip away too quickly are not unique to modern times. They appear in classical antiquity (e.g., Lucretius), in early reflections on subjective time and memory (e.g., Augustine, 4^th^-5^th^ c.), and in philosophical treatments of the human condition from the early modern period through the twentieth century (e.g., Montaigne, 16^th^ c.; Pascal, 17^th^ c.; Schopenhauer, 19^th^ c.; Camus, 20^th^ c.). We therefore prioritize accounts that apply across historical eras.

With these considerations in place, we turn first to a broad family of accounts that emphasize “cold” (i.e., purely cognitive) mechanisms shaping LTJs.

## 3 “Cold” mechanistic accounts

Table 1 provides a snapshot of established and emerging accounts of the “cold” (purely cognitive) mechanisms underlying LTJs. This section reviews each account in light of our current explanatory considerations.

**Table 1:** Overview of “cold” explanatory accounts of life tempo judgments and their empirical status.

**Account**	**Empirical tests and time scales investigated**	**Future directions**
**Ratio:** Life judged as faster with age because each year represents a smaller proportion of total lifetime.	**Indirect:** Age positively predicts judged tempo of long periods (decade) but not short periods (e.g., week; Wittmann and Lehnhoff, 2005).	Experimentally test ratio framing; explore developmental comprehension of temporal proportion.
**Internal pacemakers:** Aging slows physiological or cognitive internal clocks, making external time appear faster.	**Direct:** Faster LTJs of short and long periods (second, month, week, decade) linked to lower arousal/metabolic rate (Oshakbayev et al., 2025) and to cognitive decline (Teghil et al., 2025).	Integrate physiological markers with retrospective tempo judgments across age groups.
**Cultural acceleration:** Modern technological and social change makes life appear to pass quickly.	**Indirect:** Historical analyses linking digital technology to sense that life is fast (year, decade; Wajcman and Dodd, 2016).	Compare tempo judgments among groups exposed to varying degrees of technological change.
**Attention immersion:** Time seems to pass quickly when attention is absorbed and temporal self-monitoring is suppressed.	**Indirect:** Attentional absorption produces faster LTJs (minute, hour; Wearden, 2016). None examining boundary encoding or attentional displacement in long-period LTJs.	Test whether sustained absorption over long periods (e.g., month, year) predicts faster LTJs via reduced temporal monitoring.
**Routine compression:** Periods are retrospectively judged as having passed quicker when fewer distinct events are encoded in memory.	**Indirect:** In contextual change studies, lower recalled event complexity predicts faster LTJs (second, minute; Zakay and Block, 1997). **Direct:** Routine activity is remembered as shorter (minute, day; Avni-Babad and Ritov, 2003). “Chunking” target period's events into few categories leads to faster LTJs (year; Landau et al., 2018). Mixed evidence that remembered routine predicted faster LTJs (summer, year; Ryu et al., 2024).	Develop ecologically valid measures of everyday distinctiveness; test chunking effects longitudinally.

Parentheses following empirical findings indicate the time periods examined.

### 3.1 Ratio

A long-standing account of the impression that life seems to quicken with age is that individuals implicitly compute the ratio of a target period to their total lifetime (Janet, 1877). For example, 1 year equals one-tenth of a 10 year old's life but only one-seventieth of a seventy-year-old's.

Despite the appeal of grounding LTJs in precise mathematical relationships, this ratio account offers little empirical leverage beyond predicting a positive correlation between age and judgments of decades passing progressively faster (Draaisma, 2004; James, 1890/1950). It also struggles to explain LTJs about short periods, such a month (although, as noted, cross-scale applicability is not a necessary condition for explanatory adequacy).

### 3.2 Internal pacemakers

Researchers have shown that physiological changes with age (e.g., heart-rate variability, arousal) slow one's internal clock, making external time appear to pass more quickly (Draaisma, 2004; Oshakbayev et al., 2025). Related evidence shows that age-related cognitive decline predicts faster LTJs of long life intervals (Teghil et al., 2025).

Such accounts align with our definition of LTJs as comparative evaluations of a target period's felt duration with a subjective standard for what periods of that length are expected to feel like. These accounts furthermore precisely define what those standards are (internal “ticks”) and how those standards are instantiated in physiological processes. Highly generative and historically generalizable, internal pacemaker accounts hold significant promise for explicating LTJs.

One qualification: they do not appear suitable for explaining how people judge the tempo of shorter autobiographical periods, such as their past month or semester. As we emphasized earlier, however, cross-scale applicability should not be treated as a necessary requirement for explanatory adequacy.

### 3.3 Cultural acceleration

Several scholars in the humanities treat the sense that life moves quickly as a symptom of cultural modernity and its oft-cited pathologies—technological change, multitasking, and a constant influx of stimuli (Rosa, 2015; Wajcman and Dodd, 2016).

However intuitive, this account has received scant direct testing. Indeed, it may struggle with respect to our generativity consideration, as different specifications of mediating mechanisms vary in their empirical tractability. To illustrate, we might unpack the culture-LTJ link by proposing that socialization in, and sustained engagement with, a fast-paced culture engenders a global feeling of haste, which colors how members of that culture evaluate their personal timeline. Although this may be true, it would be impossible to randomly assign individuals to such conditions. A related possibility is that people carry an internalized standard for the number and concentration of remarkable cultural events that take place within a lifetime, and modern (vs. pre-modern) individuals metaphorically check those boxes progressively faster. Experimental or correlational tests of this possibility would have to operationalize those internalized standards.

Arguably, the mechanistic account most commonly reflected in qualitative treatments of cultural acceleration is this: individuals socialized in a modern context of capitalism and neoliberalism are expected to organize their lifestyle and identity around work and continued self-improvement, and thus feel historically unprecedented pressure to conform to norms of time optimization (Levine, 1997; Odell, 2023). This account is generative, yielding testable predictions about the effects of salient cultural norms and associated feelings of time pressure on LTJs. Indeed, later we'll review some preliminary evidence for this possibility. Still, pursuing this mechanistic account requires incorporating social-psychological insights into the motivational factors driving conformity to cultural norms, and might therefore occasion us to re-classify the culture-LTJ link as a motivational process rather than a “cold” (passive) consequence of enculturation.

Depending on how researchers specify its mediating processes, the cultural acceleration account also raises concerns about cross-scale applicability and historical generalizability. The broad impression that everything is happening faster these days appears well-suited to explain why, for example, one's seventh decade appears shorter than one's first decade, but it does not seem capable of explaining why, within the same cultural context, last weekend was over in a flash. Further, the historical ubiquity of “too fast” complaints suggests that the phenomenon predates and transcends modern sociocultural conditions.

### 3.4 Attention immersion

An established finding in the time perception literature is that when people spend minutes or hours deeply absorbed in an activity, they later judge that interval as having passed quickly, whereas low attention engagement states such as boredom or waiting make the same interval seem slow in retrospect (Burdick, 2017; Csikszentmihalyi, 1990; Wearden, 2016; Zakay, 2014). This finding is often summarized in the adage that *time flies when you're having fun*, although this formulation overlooks the fact that the effect is found with any attention-immersive activity, even those that are not “fun.”

The current understanding attributes this effect to varying awareness of time itself. When attention is directed toward non-temporal aspects of experience, fewer cognitive resources are allocated to monitoring time, and the interval later feels fast (Johnson and MacKay, 2019). Conversely, when attention drifts to time's passage, the interval seems drawn out.

This effect is exemplified in *flow* (Csikszentmihalyi, 1990), the state of deep absorption in a task that is optimally calibrated to one's skills—not so easy as to be boring, not so difficult as to provoke anxiety. Studies show that people report losing their sense of time while running, painting, or reading, only to be surprised afterward by how much time has elapsed.

Although robust at short timescales, this attention-immersion mechanism appears ill-suited to LTJs regarding extended autobiographical periods. Sustained absorptive engagement, and thus suspended temporal awareness, across, say, a 5 year period seems rare, and even if possible, it would be difficult to disentangle attentional processes from changes in memory, motivation, and meaning. As we conceded earlier, however, it may ultimately prove that qualitatively distinct processes underlie LTJs at different temporal scales, and a variant of the attention immersion account may continue to shed significant light on judgments of short periods.

### 3.5 Routine compression

In his seminal analysis of time perception, William James (1890/1950) attributed the apparent speeding up of the years with old age to

“…the monotony of memory's content, and the consequent simplification of the backward-glancing view. In youth we may have an absolutely new experience, subjective or objective, every hour of the day. Apprehension is vivid, retentiveness strong, and our recollections of that time, like those of a time spent in rapid and interesting travel, are of something intricate, multitudinous, and long-drawn out. But as each passing year converts some of this experience into automatic routine which we hardly note at all, the days and the weeks smooth themselves out in recollection to contentless units, and the years grow hollow and collapse.” (p. 625)

Since James, psychologists, philosophers, and novelists alike have emphasized routine's role in “too fast” LTJs (Fraisse, 1963; Guyau, 1890; Hofstadter, 2001; Knausgaard, 2013; Mann, 1996; Wittmann, 2016). Routine is also the main culprit in the popular imagination: when Lee and Janssen (2019) asked people what causes the impression that life speeds up with age, the most common answer was that life becomes progressively more routine.

Formalizing James's insight, events interpreted as *non*-routine—novel, demanding, or unexpected—are encoded in autobiographical memory as richly detailed episodes. Consequently, when one reconstructs a minimally routinized period, numerous distinct events stand out (Conway and Pleydell-Pearce, 2000). The period appears to expand to accommodate those events, creating the perception of its length.

By contrast, during a routinized period, few changes or events are encoded. In the language of action-identification theory (Vallacher and Wegner, 2012), routine induces a shift from concrete to abstract action representations in memory (e.g., multiple actions involved in running familiar errands are subsumed under the abstract label *running errands*). Similarly, Hofstadter (2001) proposed that routine leads people to downplay the distinctive features of analogous events and bundle them into broader *chunks*, that is, generic semantic categories such as *commute, work, entertainment*, or *family time*. Recalling fewer distinct events makes a period appear shorter—and thus faster—in retrospect.

To illustrate, consider Claire, who relocates across the country to attend college. Her first semester is anything but routinized. Each day she encounters novel stimuli and situations that demand problem-solving, decision-making, goal setting, and self-regulation. Upon reflection, Claire recalls distinct, varied events, such as discovering her favorite Thai restaurant, developing new study habits, and struggling to find a good OBGYN.

Fast-forward to her senior year, and her once dynamic college life has become routinized. In the same semester-long interval, events that would have previously occupied separate slots in memory are now compressed into generic categories: “What did I do this semester? School, hung out with friends…that's about it.” The semester “shrinks,” producing a sense of brevity.

Although the term “routine” often carries negative or pejorative connotations, routine compression is fundamentally a “cold,” purely cognitive account. It describes an implicit use of a conceptual metaphor that people ordinarily use to conceptualize abstract ideas about time in terms of relatively more concrete ideas about physical containers that vary in their *fullness* and thus *length* (Lakoff and Johnson, 1980). Influential theoretical presentations of routine compression feature related metaphoric terms such as “internal optics” (Guyau, 1890) and “telescoping” (Friedman and Janssen, 2010) to invoke the eye's spatial perspective: a routinized period containing few memorable events appears *empty* and *shrinks* or *collapses*, whereas one *filled* with many distinct memories *stretches* or *expands*.

This routine compression mechanism is often described as a two-step process: routine → fewer recalled events; fewer recalled events → faster LTJs. Here, however, we decompose it into three component associations to reflect how related hypotheses have been operationalized and tested across the empirical literature. We review these links sequentially for clarity, without presuming a fixed causal ordering.

#### 3.5.1 Routine → judged tempo

Higher levels of routine during a target period are expected to predict a faster LTJ of that period.

Studies investigating this association have yielded inconclusive results. (Avni-Babad and Ritov 2003) reported two field studies showing that people remembered periods filled with routine (vs. non-routine) activities as passing faster. For example, members of a kibbutz reported that time spent in a routine job passed more quickly than time spent in a non-routine one. Although activity type is an indirect indicator of subjective routine, it offers a concrete behavioral anchor and thus an ecologically valuable starting point for examining how daily structure shapes judged tempo.

Winkler et al. (2017) measured routine directly by means of self-reports and found that participants' ratings of routine in their “current life situation” were associated with faster LTJ of their ongoing activity and their last 5 years. In a similar study, Friedman and Janssen (2010) asked participants to rate the level of routine vs. novelty in their “recent years.” Unlike Winkler et al., they found no relation between self-reported routine and LTJs for long or short periods.

More direct tests of this association require assessing both routine and LTJs with reference to the same past period. Wittmann et al. (2015) asked participants to make broad, retrospective judgments about both routine and tempo of their past decade, yet they found no correlation between those measures. Ryu et al. (2024), across four correlational studies, assessed routine by asking participants to think back on either their past year or past summer and indicate their agreement with statements such as “During this period, life was pretty much the same from day to day.” Participants also judged the tempo of the same period using three face-valid items adapted from established scales (Friedman and Janssen, 2010; Janssen et al., 2013; Landau et al., 2018; Wittmann and Lehnhoff, 2005). Remembered routine predicted faster judged tempo in only half the samples, yielding mixed support for the hypothesized association.

#### 3.5.2 Routine → event number

The second theoretically derived prediction is that the more routinized a target period is recalled to be, the fewer memorable events stand out from that period. To our knowledge, only one empirical publication has addressed this association, which is surprising given the prominence of the event number construct in theoretical treatments of routine compression. Ryu et al. (2024) developed a self-report measure of event number by asking participants to reflect on a past period (summer or year) and indicate how many events stood out from that period, regardless of valence or objective importance. Responses were provided on a sliding scale from 0 to 100. Remembered routine predicted event number in the hypothesized direction in only one of four studies, offering limited support for this association.

Although not directly addressing routine, Teghil et al. (2025) found that the number of events recalled from the past year or decade decreased with age. This pattern may not necessarily reflect greater routine, as perceived significance of these periods increased with age.

#### 3.5.3 Event number → judged tempo

The third theoretically derived prediction is that event number will predict slower LTJs. Cognitive research inspired by the contextual-change model (Zakay and Block, 1997) has amassed a substantial body of evidence bearing on this association. According to this model, people use the number of contextual changes introduced during a period as a scaffold for estimating its duration: the fewer changes or recalled events, the shorter the period appears in retrospect. Supporting studies show that people judged a period as having passed faster when they spent it performing tasks that were predictable rather than unpredictable (Avni-Babad and Ritov, 2003; Zakay et al., 1994), affectively dull rather than arousing (Droit-Volet et al., 2004; Kim and Zauberman, 2013), and easy rather than effortful (Vohs and Schmeichel, 2003).

Although these experimental studies offer valuable insight, they focus on tempo judgments on the scale of seconds and minutes, providing only indirect evidence for the event-number/judged tempo association on the scale of extended lived periods. A few studies have examined event number or density at that scale. Zauberman et al. (2010) demonstrated that when participants were prompted to recall a greater number of event markers associated with an event, the event was judged as having occurred longer ago—indirectly suggesting that autobiographically dense memories create the phenomenological experience of the period expanding.

Landau et al. (2018) provided a more direct test by experimentally manipulating the extent to which people “chunked” their past year—Hofstadter's (2001) term for mentally grouping individual events into generic categories. Across three studies, participants led to chunk (vs. not chunk) their past year assessed it as passing faster.

Ryu et al. (2024) conceptually replicated this work using correlational methods. Across four studies, event number either failed to predict judged tempo (Study 2) or predicted it in the *opposite* direction: more memorable events, faster LTJs (Studies 1, 3, and 4). Similarly, Kosak et al. (2019) and Teghil et al. (2025) reported no correlation between event number and judged tempo.

Summing up, the notion that routine makes life appear fast is conventional wisdom and prominent theorists have formalized this notion into an elegant mechanistic model comprising three linked associations. And yet, empirical support remains surprisingly thin.

Some of this inconsistency may reflect methodological limitations: manipulating routine over long intervals is impractical (and potentially unethical), and retrospective self-reports may provide only coarse measures of lived routine. Perhaps the most robust findings—those using activity type as a proxy (Avni-Babad and Ritov, 2003)—suggest that future research should capture routine naturalistically rather than through global self-ratings.

Another concern is the poor showing of event number, the construct theorized to mediate routine's quickening influence. The few relevant studies paint a mixed picture. Landau et al. (2018) found that “chunking” a past period produced faster tempo judgments, yet studies using direct measures of event number reported negligible or even opposite event number-tempo correlations (Ryu et al., 2024; Teghil et al., 2025). These findings raise several possibilities to be explored. Perhaps event number contributes little to LTJs. Perhaps existing measures have failed to capture the relevant form of experiential differentiation. Ryu et al.'s measure may capture only “major” events (e.g., vacations, serious illnesses), whereas theory concerns the erosion of ordinary experiential distinctiveness—what James called experiences arriving “every hour of the day.” An important challenge for future work will be developing methods that capture more granular experiential flattening.

As we alluded to, routine compression is a quintessential “cold” account, reducing LTJs to a function of the *quantity* of events recalled within a target period. And yet, a closer look at the theorizing reveals an affective undercurrent, a sense that fast life periods are those that somehow failed to meet one's expectations for what experience should be. This tone can be heard in the earlier James quote, and it rings out in the theorist Guyau's (1890) reflection:

“Old age…is more like the unchanging scenery of the classical theater, a simple place, sometimes a true unity of time, place and action that concentrates everything round one dominant activity and expunges the rest; at other times the absence of time, place and action. The weeks resemble one another, the months resemble one another; the monotony of life drags on. All these images fuse into a single image. In the imagination, time is abridged. Desire does the same: as we approach the end of life we say every year: ‘Another year gone! What did I do with it? What did I feel, see, achieve? How is it possible for the three hundred and sixty-five days that have passed to seem no more than a couple of months?' ” (quoted in Draaisma, 2004, p. 207)

This affective undercurrent suggests that “hot” mechanisms, like compensating for dissatisfaction, may also shape LTJs, a possibility we consider next.

## 4 “Hot” mechanistic accounts

Complementing our review of “cold” mechanistic accounts, another family of established and emerging proposals focuses on “hot” mechanisms—those charged with affect or motivation. These accounts are summarized in Table 2 and reviewed in this section.

**Table 2:** Overview of “hot” explanatory accounts of life tempo judgments and their empirical status.

**Account**	**Empirical tests and time scales investigated**	**Future directions**
**Growth deprivation:** Life feels faster when intrinsic growth needs are unfulfilled, making time seem wasted.	**Indirect:** Current time pressure predicts faster LTJs (week, month, year; Janssen et al., 2013; Wittmann, 2016). **Direct:** Positive, not negative, growth–tempo correlations (summer, year), contrary to predictions (Ryu et al., 2024).	Manipulate remembered growth vs. stagnation; test nonlinear relationships between growth, satisfaction, and tempo.
**Performed speed:** Reporting that time passes quickly serves as a socially normative way to convey busyness, competence, and personal worth.	**Indirect:** Familiarity with the folk belief that “life speeds up with age” predicts faster LTJs (year; Lee and Janssen, 2019). Retrospective duration judgments shift toward group consensus in a task that lasted several dozen minutes (Conway, 2004).	Experimentally manipulate productivity or time-optimization norms; test whether reminders of leisure stigma or moral industriousness shift LTJs.
**Longing:** Cherished, personally significant periods appear fleeting because their value alters temporal judgment—either through affective scarcity (precious/irretrievable) or motivational proximity (brought metaphorically closer to afform positive self-evaluations).	**Indirect:** Self-enhancing past periods (in months, years) are judged as more recent (Ross and Wilson, 2002). General preference for nostalgia predicts faster LTJs (year; Landau et al., 2018). **Direct:** Nostalgic longing for a target period predicts faster tempo judgment (summer, year; Ryu et al., 2024).	Manipulate affective contrast or self-esteem relevance of recalled life periods to test whether scarcity or motivational proximity compress intervening time.

Parentheses following empirical findings indicate the time periods examined.

### 4.1 Growth deprivation

Routine compression addresses how many events are remembered in a target period, but it does not model how people evaluate what they “got out of” that period—the perceived value or meaning of those experiences—and how such evaluations might shape comparative judgments of duration.

Among motivational accounts of LTJs, the idea that time feels fast when it was unfulfilling is arguably the most historically entrenched and intuitively compelling starting point. The ancient philosopher Seneca (2005) counseled that life only appears short when it is squandered in pursuit of extrinsically motivated goals such as fortune and fame. A similar sentiment was echoed centuries later by the French moralist La Bruyère and de (1688), who wrote, “Those who make the worst use of their time are the first to complain of its brevity.”

To distill and formalize this notion, we draw on self-determination theory (SDT; Deci and Ryan, 2000), a widely researched motivational theory in social psychology. SDT posits that people are inherently motivated to *grow*—to explore, master challenges, and integrate experience into a coherent sense of self. Circumstances and activities that foster growth satisfy three basic psychological needs: autonomy (a sense of choice in what one does), competence (feeling effective in what one does), and relatedness (feeling meaningfully connected with others). When these needs are satisfied, people experience their actions as self-determined; when they are thwarted, people see their actions as less intrinsically satisfying and more externally controlled (e.g., by social approval or obligation).

Research inspired by SDT shows that growth motivation strongly influences satisfaction with one's experiences (Deci and Ryan, 2002). Individuals in marriages, academic settings, or weight-loss programs who perceive opportunities for growth report greater satisfaction and wellbeing than those who feel deprived of growth opportunities. For example, law school students who felt that faculty supported (vs. thwarted) their autonomy reported higher life satisfaction (Sheldon and Krieger, 2007).

We can apply SDT as a framework for explaining how growth motivation informs LTJs. It is possible that individuals evaluate a past period as “their own” when they interpret it as affording opportunities for growth—when they could freely choose actions that align with their intrinsic interests and fulfill their psychological needs. Thus, when reflecting on a period seen as rich in growth, individuals will be satisfied with how they spent their time, and they will be less likely to judge that time as having passed too quickly. When, in contrast, they reflect on a period during which they felt deprived of growth, they will view that period as unsatisfactory and brief, feeling it passed too quickly without meaningful engagement. On this view, LTJs serve not merely as memory-based reconstructions, but as evaluative summaries of whether time was experienced as well spent.

To illustrate this *growth deprivation* account, imagine Cory, who remembers his past month as dominated by externally imposed activities—filling out graduate school applications to meet parental expectations and working a menial job. With few opportunities to explore, improve skills, or connect with others in intrinsically satisfying ways, Cory experiences this period as lost or wasted time.

The growth deprivation mechanism is less a challenger to routine compression than a formalization of the evaluative “loss” theme that quietly runs throughout it. In this sense, it supplements an otherwise “cold” cognitive narrative with an explicit motivational evaluation. Through this theoretical lens, people conceptualize time metaphorically not in terms of spatial expansion and contraction, but in terms of a scarce commodity that can be squandered. This account applies equally well to short and long periods, from days to decades. As to historical generalizability, it explains age-old life tempo laments as well as potential exacerbation via the modern-day hamster wheel of extrinsically controlled responsibilities.

If this account captures a core motivational truth about how people evaluate lived time, then remembered growth during a period should be inversely related to judged tempo for the same period. A more specific prediction is that low remembered growth will predict lower global satisfaction with the period, which will in turn predict faster judged tempo.

Partial support for these predictions comes from studies showing that felt time pressure correlates with faster LTJs. For example, individuals who report feeling busy or rushed tend to judge past weeks, months, and years as having passed quickly (Friedman and Janssen, 2010; Janssen et al., 2013; Joubert, 1984; Wittmann and Mella, 2021; Wittmann et al., 2015). Although not explicitly focused on LTJs, SDT research shows that felt time pressure positively correlates with extrinsic control over one's actions and limited opportunities for self-determined growth (Deci and Ryan, 2016).

Still, time pressure is only an indirect proxy of perceived growth deprivation. Moreover, these studies typically assess time pressure in the present, whereas the growth deprivation account focuses squarely on the relationship between remembered growth affordances and judged tempo for the same past period.

Ryu et al. (2024) directly tested the correlations predicted by the growth deprivation account. Using an adapted version of Anderson et al.'s (2020) Personal Growth and Development Scale, they had participants rate the extent to which their experiences during the past year or past summer provided growth-promotive gains in autonomy (e.g., “gain the strength to stand up for what I believe”), competence (e.g., “take advantage of opportunities in my surroundings”), and relatedness (e.g., “learn how to develop meaningful relationships with others”). Participants also reported their global satisfaction with the target period (e.g., “If I had this past year to live over, I would change nothing”) and their judgment of how fast the period appeared to pass.

Again, the SDT-inspired growth deprivation account predicts that periods lacking opportunities for growth will feel unsatisfying and fleeting. Yet across four studies utilizing demographically diverse samples, results consistently revealed the *opposite* pattern: People who felt they had grown *more* during a period, and who were more satisfied with it, judged that time as having passed faster. This recurring pattern, though not definitive, is striking because it reverses the expected relationship between fulfillment and LTJs.

This pattern is not entirely without precedent. Baum et al. (1984) found that older adults engaged in autonomy-building activities and who reported a strong sense of purpose in life tended to judge recent time as passing faster than counterparts with depressive symptoms. Relatedly, events experienced as positive (vs. negative) are judged to have passed more quickly (Cui et al., 2023; Pollatos et al., 2014).

Why, then, does fulfillment—rather than deprivation—appear to predict “too fast” LTJs? To make sense of this pattern and to guide future theoretical and empirical work, we next present two motivational accounts, each offering a distinct interpretive framework for the positive growth/tempo correlation.

### 4.2 Performed speed

The accounts considered thus far all begin from the premise that the phenomenon to be explained is a subjective impression that a life period passed more quickly than expected. But perhaps much of what people report as “time flying” reflects something different: *performed* speed, or the act of *signaling* temporal scarcity.

Why signal temporal scarcity at all? Because there is a widespread social norm to be, or at least appear, busy. Scholars across disciplines have described how modern productivity culture equates busyness with personal worth (Lauer, 1981; Levine, 1997; Odell, 2023). Correspondingly, harboring “dead time” carries stigma, and unstructured leisure time is morally ambiguous, especially in the modern West (Odell, 2023). Although cross-national and regional differences exist in how people prioritize efficient goal pursuit (Levine, 1997; Lowin et al., 1971; Triandis, 1994), most individuals alive today experience normative pressure to organize life around synchronized clock time and, under the lingering influence of the Protestant work ethic, to maximize productivity.

Combine that insight with decades of social-psychological research showing that people are motivated to sustain a culturally valued self-image and to convey that image publicly. For example, self-presentation theory identifies self-promotion—advertising one's achievements or industriousness—as a common strategy to appear competent (Jones and Pittman, 1982).

Integrating these literatures raises the possibility that *saying* time flies functions to signal busyness and temporal scarcity as indicators of culturally valued competence. Conversely, to publicly report—or, through repeated self-presentation, even come to believe—that one's summer, year, or decade passed at a comfortable pace would risk implying a lapse in norms of time optimization.

This *performed speed* account offers a potential reinterpretation of the consistent positive correlation between remembered growth and judged tempo observed by Ryu et al. (2024). In those studies, “growth” was operationalized as a summary evaluation of gains in competence, autonomy, and relatedness. Yet such retrospective appraisals may be confounded by motivated preferences to view one's experiences and activities as culturally sanctioned, productive, and self-actualizing. The positive growth–tempo correlation may therefore reflect a third variable: the motivation to see oneself as “living their best life” and to project that identity by reporting that time is insufficient to accommodate their self-actualization.

Some findings are consistent with the notion that social norms and self-presentation motives influence tempo judgments. Conway (2004) found that retrospective duration judgments were influenced by conformity to group opinion, suggesting that retrospective time judgments are, at least in part, socially calibrated. Similarly, Lee and Janssen (2019) showed that participants who were familiar with the folk notion that “life speeds up with age” were more likely to report that time passed quickly for them personally—precisely the pattern a performed speed account would predict.

To our knowledge, no research has directly tested whether beliefs or expressions of “too fast” LTJs function as self-presentational signals. One approach would be to manipulate the salience of productivity or time-optimization norms and assess corresponding changes in LTJs.

This account may, however, fall short of the theoretical criterion of historical generalizability established earlier. It locates LTJs (at least in part) within distinctly modern efforts to signal compliance with cultural norms of productivity and to avoid the stigma of unoptimized time. Yet it remains possible that this motive has older antecedents in a more general desire to project moral industriousness and control over one's time.

### 4.3 Longing

Growth deprivation theorizing attempts to capture dissatisfaction with time that felt unfulfilling. Anecdotally, however, the periods people most treasure are also those they describe as having passed too quickly. This enduring lament suggests a mechanism of *longing*: an emotional or motivational “pull” toward a cherished period.

Both deprivation and longing are theorized to influence LTJs by appraising past periods against ideals of fulfillment, yet they diverge in emotional tone: deprivation laments missed opportunity; longing mourns abundance already spent. We consider two complementary but theoretically distinct processes by which longing might shape LTJs—one affective, tied to scarcity, and one motivational, tied to self-appraisal.

#### 4.3.1 Longing as scarcity

Personally cherished periods—those imbued with purpose, joy, or connection—may be experienced as rare commodities: fleeting, precious, and irretrievable, especially when contrasted with drab or externally constrained current circumstances. Individuals may regard such periods as they regard scarce resources in other domains, valuing them because they are rare and unrecoverable. When individuals lament that “those were the best years of my life, and they flew by,” they may be expressing an affective logic rather than a perceptual illusion: the more meaningful and fulfilling a period, the more one mourns its finitude. The same logic can apply to shorter intervals: “the weekend flew by” may reflect the belief that the weekend was deeply enjoyable and is now irretrievably gone. The assessment that a period passed quickly, in this view, is a byproduct of loss awareness and longing for fulfilling times.

Indirect evidence comes from studies linking nostalgia, a sentimental longing for a past episode or period, to growth as defined by SDT. People report being nostalgic for times that follow a redemptive pattern (Wildschut et al., 2006), strengthened social connection (Sedikides et al., 2016), and during which they felt free to act in alignment with intrinsic values (Baldwin et al., 2015; Kelley et al., 2022), resonating with SDT's emphasis on, respectively, competence, relatedness, and autonomy.

Is nostalgic longing linked to LTJs? Landau et al. (2018) found that a general preference for nostalgic reverie correlated positively with faster tempo judgments for one's past year, though they did not assess nostalgia and judged tempo for the same period. Ryu et al. (2024) addressed this, reporting positive correlations between remembered growth and nostalgia, as well as between nostalgia and judged tempo, within the same periods (Studies 3 & 4). Although more direct causal tests are required, these preliminary findings are consistent with the idea that growth-abundant periods appear to fly by because they are experienced as scarce—and thus fleeting—opportunities for self-actualization.

Still, nostalgia remains an indirect proxy for the key scarcity construct, which has yet to be directly assessed. Future research could test this possibility by manipulating affective contrast between a cherished and a mundane period and examining whether increased felt scarcity predicts faster LTJs.

#### 4.3.2 Longing as self-appraisal

If scarcity explains longing's affective side, self-appraisal may explain its motivational side. According to self-appraisal theory (Wilson and Ross, 2001), people strategically construct the spatiotemporal distance of past episodes to maintain a favorable view of their current self. Past episodes that reflect positively on one's current self are metaphorically brought closer (“it feels like yesterday”), whereas less flattering times are pushed away (“that's ancient history”). Supporting studies show that remembered events that bolster one's self-esteem are judged as more recent, and thus spatially closer to one's current self-concept, compared with unflattering events from the same time (Ross and Wilson, 2002).

Applied to explain LTJs, this framework suggests that growth-rich periods—those in which people saw themselves as competent, loved, or alive—may appear not only emotionally salient but also metaphorically spatially near. Mentally “pulling” those periods closer to one's current self-concept may *collapse the intervening time*, producing the impression that it passed quickly. To illustrate, the feeling that “college feels like it was just yesterday” may reflect motivated proximity of past to current self, which “scrunches” the time between then and now.

In this speculative account, the experience of life tempo arises not from deprivation but from identification: the drive to preserve continuity with a valued self-image. This would explain the positive growth–tempo correlation observed by Ryu et al. (2024): by mentally pulling a growth-rich period near one's current self, the intervening interval seems short.

Future studies could test this possibility by asking participants to recall self-enhancing vs. self-diminishing (or neutral) periods, estimate the subjective distance between their current self-concept and those events, and rate the duration of the intervening time. Self-enhancing recalls should uniquely compress estimated temporal distance relative to neutral or self-diminishing periods.

Together, these two “longing” accounts suggest that longing's temporal signature differs from deprivation's not in kind, but in direction. Both involve evaluating the yield of lived time against internal standards, yet deprivation laments what was missing, whereas longing mourns what was meaningful. In both cases, the affective logic of value translates into the phenomenology of quickness.

## 5 Taking stock, looking ahead

Two millennia of theorizing about the pace of lived time—spanning cognitive, motivational, affective, cultural, and social perspectives—converge on a central insight: LTJs reflect not only what happened during a period, but how those events were experienced, encoded, and interpreted.

In this article, we reviewed major theoretical accounts and emerging proposals (summarized in Tables 1, 2). No single account fully explains the phenomenon; instead, each marks a distinct coordinate on the emerging map, which we offer as a conceptual scaffold for future theoretical refinement and empirical testing.

A clear direction for future work is to probe individual mechanisms more deeply, resolving mixed findings, sharpening measurement, and pursuing direct tests where only indirect ones currently exist. Perhaps the most striking regularity across the reviewed evidence is that periods remembered as most meaningful are also those often described as fleeting. This pattern falls outside the explanatory scope of routine compression (at least when formalized strictly as a function of event quantity) and contradicts the intuitive prediction of a growth deprivation account, which would expect *unfulfilling* periods to “zip by.”

One illustrative case for methodological refinement concerns the operational definition of *eventfulness*. In the routine compression framework, a past period remembered as eventful is assumed to appear subjectively “larger” and thus slower in tempo. Implicit in this view is a spatial metaphor of time as a physical container that expands or contracts depending on its contents.

An alternative and intuitive possibility is that, when participants are asked to reflect on a period's eventfulness, they may instead conceptualize time metaphorically as a path along which the self moves forward (for evidence that *journey* metaphors shape conceptions of the self in time, see Landau et al., 2014). Along this path, one passes culturally defined autobiographical milestones (e.g., achievements, purchases, skills). Within this construal, an eventful period may appear fast in retrospect because the self is perceived as having passed many milestones.

From this perspective, the relation between eventfulness and LTJs may depend on which spatial metaphor is brought to bear when construing a past period. When time is construed as a container, greater remembered eventfulness may be associated with slower LTJs (eventful = full). When time is construed as a path, greater remembered eventfulness may instead be associated with faster LTJs (eventful = much ground traversed). Clarifying how participants interpret questions about eventfulness and pace may therefore help explain why empirical findings linking eventfulness to LTJs sometimes diverge in direction.

Beyond refining individual accounts and methods, future work should examine how multiple mechanisms intersect in shaping LTJs. These processes are unlikely to operate in isolation. For example, routine may limit opportunities for growth, amplifying feelings of deprivation; longing may emerge when periods of absorption give way to reflective distance, making cherished intervals feel doubly fleeting; and temporal compression may become distressing primarily when it is evaluated as a loss of meaning rather than experienced as neutral efficiency. In addition, cultural scripts that valorize busyness and stigmatize leisure may amplify the judgment that a period passed quickly independently of autobiographical memory processes.

Importantly, these mechanisms may also vary in their relevance across temporal scales and life stages. Compression driven by routine may be especially salient when judging relatively short or repetitive intervals (e.g., weeks or months), whereas longing or other affectively-charged comparisons may play a larger role in judgments of extended life chapters such as decades, particularly during later-life review (Butler, 1963). More generally, it remains an open question whether the same processes govern LTJs for short vs. long autobiographical periods. Judging the pace of one's past week may rely more heavily on attention, time pressure, or density of experience, whereas judging the pace of a decade may depend more on reflective comparison, aspiration, or counterfactual evaluation.

This possibility challenges the cross-scale applicability consideration outlined earlier. Although it may be appealing to identify a single explanation that spans judgments of weeks, years, and decades, a pluralistic or mosaic model—one in which different mechanisms dominate at different scales—may ultimately provide a more accurate account of how people evaluate the passage of their lives.

The remembered pace of life has been linked to a range of consequential outcomes, including global life satisfaction, death anxiety, and long-term goal pursuit (Carstensen, 2006; Quinn and Reznikoff, 1985; Wittmann, 2016). For this reason, research on LTJs carries potential translational relevance for understanding wellbeing. Importantly, however, LTJs do not constitute a unitary psychological construct, nor do we suggest that they represent a direct target for intervention. Across the accounts reviewed here, LTJs are best understood as subjective outcomes that emerge downstream of other psychological processes, such as engagement, motivation, and meaning.

Viewed in this light, the present review raises questions about several widely endorsed prescriptions for alleviating the distress associated with life's apparent speed. Conventional advice often emphasizes seeking novelty, increasing enjoyment, or cultivating mindfulness, on the assumption that breaking routine or enriching experience will slow the felt passage of time. These intuitions align naturally with the routine compression account, which attributes “too fast” LTJs to dull familiarity, and with growth-based accounts that equate enjoyment with intrinsically satisfying experience.

Yet, as we have seen, the empirical foundation for these prescriptions is mixed. Evidence for routine compression is weaker than commonly assumed, and findings from our own work, along with converging evidence elsewhere, indicate that remembered growth and fulfillment are often associated with faster, not slower, LTJs (Ryu et al., 2024). If novelty, enjoyment, and intrinsic motivation promote personal growth—as SDT would predict—then such experiences may paradoxically be linked to a faster remembered pace of life.

A similar ambiguity characterizes mindfulness-based approaches. Although some findings suggest that attentional engagement can lengthen judged duration in specific contexts (Hanley et al., 2015), other work indicates that mindfulness or absorption may shorten retrospective duration relative to more time-focused forms of attention (Block et al., 1980; Sedlmeier et al., 2023). Taken together, these results suggest that interventions aimed at altering attention or experience may influence LTJs, but not always in the direction of slowing remembered time.

One implication of this pattern is that a fast-remembered life need not signal poor wellbeing. Several accounts reviewed here converge on the possibility that periods remembered as having passed quickly are often those marked by mastery, engagement, meaning, or longing. Rather than treating “too fast” LTJs as inherently problematic, future research may benefit from examining when and why a fast life is experienced as fulfilling vs. distressing, and how individuals interpret what a rapid pace signifies about who they are, what they value, and how they have lived.

## Footnotes

1 We thank an anonymous reviewer for helpful comments that prompted us to clarify the metaphorical status of LTJs and to sharpen our distinction between phenomenological experience and retrospective assessment.

2 We thank an anonymous reviewer for drawing our attention to this point.
